# Addressing geographic access barriers to emergency care services: a national ecologic study of hospitals in Brazil

**DOI:** 10.1186/s12939-017-0645-4

**Published:** 2017-08-22

**Authors:** Thiago Augusto Hernandes Rocha, Núbia Cristina da Silva, Pedro Vasconcelos Amaral, Allan Claudius Queiroz Barbosa, João Victor Muniz Rocha, Viviane Alvares, Dante Grapiuna de Almeida, Elaine Thumé, Erika Bárbara Abreu Fonseca Thomaz, Rejane Christine de Sousa Queiroz, Marta Rovery de Souza, Adriana Lein, Daniel Paulino Lopes, Catherine A. Staton, João Ricardo Nickenig Vissoci, Luiz Augusto Facchini

**Affiliations:** 1 Center of post-graduate and Research in Administration, School of Economics, Federal University of Minas Gerais, Belo Horizonte, Minas Gerais Brazil; 2 Observatory of Human Resources in Health, Faculty of Economics, Federal University of Minas Gerais, Belo Horizonte, Minas Gerais Brazil; 3 Centre for Development and Regional Planning, Federal University of Minas Gerais, Belo Horizonte, Minas Gerais Brazil; 4 Department of Administrative Sciences, Faculty of Economics, Federal University of Minas Gerais, Belo Horizonte, Minas Gerais Brazil; 5 National School of Public Health, Nova University, Lisbon, Portugal; 6 Medomai information technology, Belo Horizonte, Minas Gerais Brazil; 7 Department of Collective Health, Faculty of Nursing, Federal University of Pelotas, Pelotas, Rio Grande do Sul Brazil; 8 Department of Public Health, Federal University of Maranhão, São Luís, Maranhão Brazil; 9 Department of Public Health, Federal University of Goiás, Goiânia, Goiás Brazil; 10 Division of Emergency Medicine, Duke University Health System, Duke Global Health Institute, Duke University, Durham, USA; 11 Department of Applied Social Sciences, Federal Center of Technological Education CEFET-MG, Belo Horizonte, Minas Gerais Brazil; 12 Faculty of Medicine, Departament of social Medicine, Federal University of Pelotas, Pelotas, Rio Grande do Sul Brazil

**Keywords:** Spatial analysis, Hospitals, Health care evaluation mechanisms, Emergency health services, Health services accessibility, Rural hospitals, Low-volume hospitals, Spatial autocorrelation

## Abstract

**Background:**

Unequal distribution of emergency care services is a critical barrier to be overcome to assure access to emergency and surgical care. Considering this context it was objective of the present work analyze geographic access barriers to emergency care services in Brazil. A secondary aim of the study is to define possible roles to be assumed by small hospitals in the Brazilian healthcare network to overcome geographic access challenges.

**Methods:**

The present work can be classified as a cross-sectional ecological study. To carry out the present study, data of all 5843 Brazilian hospitals were categorized among high complexity centers and small hospitals. The geographical access barriers were identified through the use of two-step floating catchment area method. Once concluded the previous step an evaluation using the Getis-Ord-Gi method was performed to identify spatial clusters of municipalities with limited access to high complexity centers but well covered by well-equipped small hospitals.

**Results:**

The analysis of accessibility index of high complexity centers highlighted large portions of the country with nearly zero hospital beds by inhabitant. In contrast, it was possible observe a group of 1595 municipalities with high accessibility to small hospitals, simultaneously with a low coverage of high complexity centers. Among the 1595 municipalities with good accessibility to small hospitals, 74% (1183) were covered by small hospitals with at least 60% of minimum emergency service requirements. The spatial clusters analysis aggregated 589 municipalities with high values related to minimum emergency service requirements. Small hospitals in these 589 cities could promote the equity in access to emergency services benefiting more than eight million people.

**Conclusions:**

There is a spatial disequilibrium within the country with prominent gaps in the health care network for emergency services. Taking this challenge into consideration, small hospitals could be a possible solution and foster equity in access to emergency and surgical care. However more investments in are necessary to improve small hospitals capabilities to fill this gap.

## Background

Health system structural deficits are a major barrier to healthcare delivery, especially when affected by inadequate distribution of emergency care services (ECS). Despite its multiple understandings, at the present work, ECS was defined as a lifesaving surgical procedure. Worldwide, an estimated 45% of deaths and 36% of disability-adjusted life years could be prevented through expanded access to ECS [1]. Despite the vital role of ECS in reducing global morbidity and mortality, access barriers persist particularly among disadvantaged populations due to socioeconomic and demographic factors. These access barriers are frequently intensified in low and middle-income countries (LMIC) [1, 2].

Traditionally, literature on access to ECS focuses on social determinants of health, and demand-side barriers to health care access, such as the unaffordable costs of medical care or inadequate patient education [3]. However, in the case of ECS, the role of supply-side factors, particularly the geographical distribution of health facilities, is not well understood [4]. Many studies concentrate on travel distance to primary care facilities and its effects on health care access, patient outcomes, and the utilization of ECS [5–9]. Likewise, there are numerous studies on the distribution of ECS in relation to patient mortality and morbidity [10–13]. However in the current literature, there has yet to be a study evaluating how effectively appropriately equipped small hospitals (SH) can fill this ECS care gaps and contribute to improve access to emergency care [14–16]. Despite challenges concerning economies of scale and quality, there is an argument that SH are needed in remote regions, particularly for general health needs beyond primary care, urgent care services, and maternal-infant care in low-risk situations [2, 15].

The interest in the health service infrastructure’s spatial distribution has gained momentum in recent years [17, 18]. Spatial analysis and geographic information systems have proven to be of great utility to study the allocation and planning of services [19]. Several literature reviews describe the evolution of research in the field of health geography and its different applications in diverse countries [19–22]. These studies showed spatial analysis has been widely useful to investigate the relationship between access, geography, utilization, quality, and health indicators. Further, the literature utilizes quantitative and qualitative methods to measure potential access to health facilities, in order to assess disparities in health systems [19, 20]. For this reason, studies of this nature are essential for health managers to analyze and define strategies for the provision of health services and policy formulation [19, 20].

Considering this context the main objective of the present work was identify geographic access barriers to ECS in Brazil. The secondary goal was to define possible roles to be assumed by SH as a potential alternative to overcome access barriers. Was hypothesized that Brazilian SH could help to minimize lack of access to emergency services.

## Methods

### Study design and setting

The present work can be classified as a cross-sectional ecological study. Were analyzed data about 5843 Brazilian hospitals in three phases: 1) evaluation of the distances among SH and nearest high complexity center (HCC) within administrative regions; 2) assessment of geographical accessibility to hospital care; 3) identification of clusters of municipalities with minimal-structured SH for surgical care located in regions with low HCC accessibility.

### Setting

**Fig. 1:**
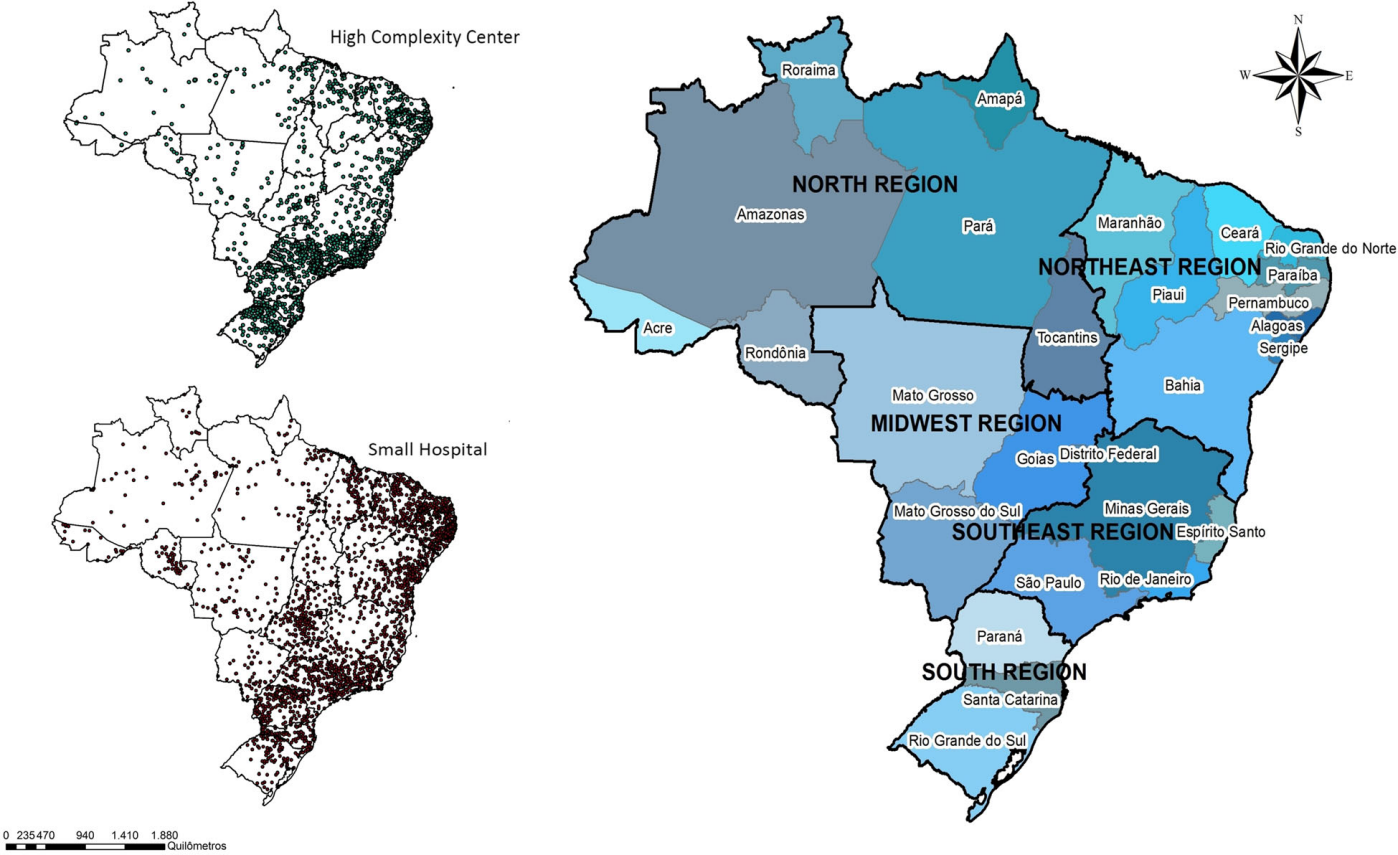
Brazilian states, regions and hospital network

Brazil is an upper-middle income country with 206 million inhabitants (IBGE, 2016). The Brazilian health system is organized to provide universal access to entire population - the Sistema Único de Saúde - SUS [23]. The country is divided into five administrative regions (North, Northeast, Center-West, Southeast and South), 26 states, and the federal district (Fig. 1). Up to 2013, the Brazilian healthcare network was composed of 5843 hospitals, 2774 of which were SH and 3069 HCC. Considering the guidelines from Brazilian Ministry of Health, a facility is classified as SH if the number of beds available is equal or less to 50. HCC are defined as institutions with: presence of certification related to ECS and capacity to offer surgical procedures related to ECS, or realized more than 1000 deliveries by month. The HCC were stratified in 2559 exclusive high complexity centers and 486 facilities that were also acting as maternities. From the universe of HCC was made the option to exclude of analysis 24 facilities for been exclusively maternities. These maternities do not act like a complete ECS facility once they do not meet all the criteria defined by Brazilian Ministry of Health [23]. In Brazil, we have guidelines trying to organize the referrals of patients, but no rule or law defining which type of health facility a municipality should have. Thus, there are municipalities in our study in all possible conditions: with SH and HCC, only SH, only HCC, none hospital.

### Data sources and variables

#### Hospital data

SH data was collected through a national survey about infrastructure and quality of care described elsewhere [24]. SH data used were: number of beds, physical structure available to offer emergency care and geographical coordinates. HCC data was collected using secondary databases to gather information about: geographical coordinates, certifications, number of beds and administrative information through the records of the National Registry of Health Facilities (NRHF or CNES in Portuguese) [23]. All data referring SH and HCC were collected during 2014.

#### Demographic data

Population data of all 5565 Brazilian municipalities was collected from the Brazilian Institute of Geography and Statistics selecting the estimates referring to 2014 [25].

### Data analysis

Data analysis was divided in three steps and was conducted using Python [26] and ARCMAP 10.2 softwares [27]. The first step is independent, while the second and third steps were related.

#### First step - distance patterns

It was evaluated the Euclidean distance of SH to the nearest HCC within the same state, simulating the healthcare network flow. In this way, the search for a referral HCC was confined to each state, excluding searches for HCC in other states even, if they were located in geographical lower distance. Distance was divided into four distinct groups, based on the regulation of the Brazilian Ministry of Health of travel time to reach health care: (a) less than 60 km from the municipality of the HCC (approximately 1 h of travel time); (b) between 60 and 90 km from the HCC (approximately 1.5 h of travel time); between 90 and 120 km from the HCC (approximately 2 h of travel time) and lastly; (d) more than 120 km from the HCC (approximately more than 2 h of travel time). This analysis highlights the existing gaps in the health care network (HCN) in relation to ECS coverage and access.

#### Second step - accessibility to ECS care

To evaluate geographical accessibility to ECS care was used the two-step floating catchment area (2SFCA) method [28, 29]. With this approach it was possible assess the accessibility by the interaction of two geographic characteristics: (a) volume of available hospital beds in each hospital to a determined population within 1 h of travel distance, and (b) the proximity of services to that population, within a 60 km buffer from each municipality centroid. The 2SFCA method generated an accessibility index for each municipality in Brazil. Two 2SFCA were performed, one for SH and other for HCC. The accessibility index of each municipality was classified in two categories: below of above the average access to ECS.

In sequence, to evaluate the discrepancies in ECS care accessibility it was performed a spatial overlap among the different ECS care scenarios considering accessibility to SH and to HCC. The spatial overlap selection highlighted geographical areas where there were simultaneous categorizations as above average access to SH and below average access to HCC.

#### Third step - spatial association

Despite the fact that municipalities selected by the spatial overlap method indicate geographic regions where SH could offer an alternative to overcome access barriers, this is not enough to ensure satisfactory emergency care. SH usually face problems related to poor quality of care due to lack of minimum emergency requirements capable of guaranteeing adequate health services [30]. Taking this limitation into account was used a minimum emergency service requirements (MESR) adherence proportion for each SH. This index was elaborated in a previous work realized by BARBOSA et all, [24]. MERS index is defined by the presence of the following structure of care: emergency monitoring equipment, electrocardiogram, mechanical respirator, defibrillator, sphygmomanometer, laboratory services and diagnosis support, physicians available 24 h, anesthesiology services, and operations during 24 h a day. This adherence proportion was used as an assessment of SH capabilities to offer more complex emergency care.

To each municipality covered by a SH but not a HCC was assigned an average of MESR index. This assignment was only realized for cities selected through spatial overlap procedure. Then was used the Getis Ord Gi spatial autocorrelation test [31] to recognize spatial clusters of municipalities considering the different levels of MESR values. Getis Ord Gi statistics evaluate the spatial dependency effect of the frequency and attribute values [32]. As a result Getis Ord Gi technique highlighted spatial clusters related to different values of MERS. Hot spots indicate clusters of high values, while cold spots the opposite. Thus, it was possible to check the presence of municipalities distant from HCC even though covered by SH with minimum conditions to offer more complex emergency care.

## Results

The majority of SH in Brazil was located at Northeast region (40,34%). The Southeast region has the higher proportion of HCC (40,57%). The North region had the lowest percentage of hospitals in both categories (Table 1). Despite the difference related to number of SH the mean of beds available did not present big changes among Brazilian regions. The Brazilian average of beds by population is quite similar among SH and HCC. For the Northeast region, there were more beds per population from SH than from HCC, highlighting the local relevance of such type of hospital to access of health services.

**Table 1:** Characterization of Brazilian health care network

	Small hospitals			High Complexity Centers		
	*N* (%)	Number of beds -mean (SD)	SH Beds per 1000 people	*N* (%)	Number of beds -mean (SD)	HCC Beds per 1000 people
Center- West	375 (13.52)	24.57 (12.74)	1.64 (1.76)	204 (6.65)	112 (117.05)	2.41 (1.71)
Northeast	1119 (40.34)	26.73 (15.44)	1.74 (1.41)	821 (26.75)	103.26 (107.88)	1.63 (1.05)
North	278 (10.02)	26.71 (13.35)	1.3 (0.91)	251 (8.18)	90.47 (75.01)	1.6 (0.97)
Southeast	606 (21.85)	32.15 (16.67)	1.55 (2.09)	1245 (40.57)	139.37 (142.97)	2.11 (1.53)
South	396 (14.28)	30.61 (16.06)	3.13 (2.51)	548 (17.86)	119.18 (123.43)	3.31 (2.15)
Brazil	2774	28.17 (15.5)	1.95 (2.05)	3069	119.09 (124.34)	2.08 (1.57)

Distance from ECS or Surgical care

### Distance from ECS or surgical care

**Fig. 2:**
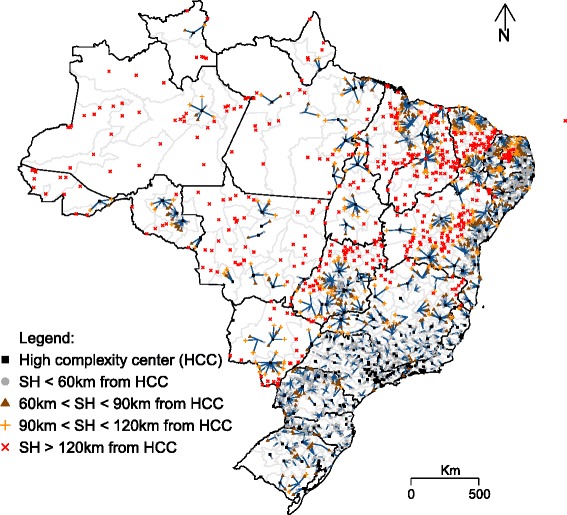
Distance among Small Hospitals (SH) and High Complexity Centers (HCC), in Brazil

In total, 432 (7,76%) Brazilian municipalities had HCC (black squares – Fig. 2). The majority of municipalities (55,99%) had SH located less than 60 km from the closest municipality with an HCC. Yet, there was still a shortage of HCC in the Central-West, rural Northeast, and especially the North regions (Fig. 2). For these regions, there was a polarization of HCC in urban centers, particularly state capitals. From this analysis, it was noted 824 municipalities (Table 2) did not meet the criteria of maximum travel time of 2 h , recommended distance by Lancet commission on Global Surgery to assure timely access to essential surgery services [24].

**Table 2:** Brazilian municipalities and distance patterns among Small Hospitals (SH) and High Complexity Centers (HCC)

	Municipalities	
	*N*	%
High Complexity Centers	432	7.76
Small Hospital in less than 60 km from high complexity center of reference	3116	55.99
Small Hospital between 60 km and 90 km from high complexity center of reference	787	14.14
Small Hospital between 90 km and 120 km from high complexity center of reference	406	7.30
Small Hospital between in more than 120 km from high complexity center of reference	824	14.81
Total	5565	100.00

### Access to ECS of surgical care

**Fig. 3:**
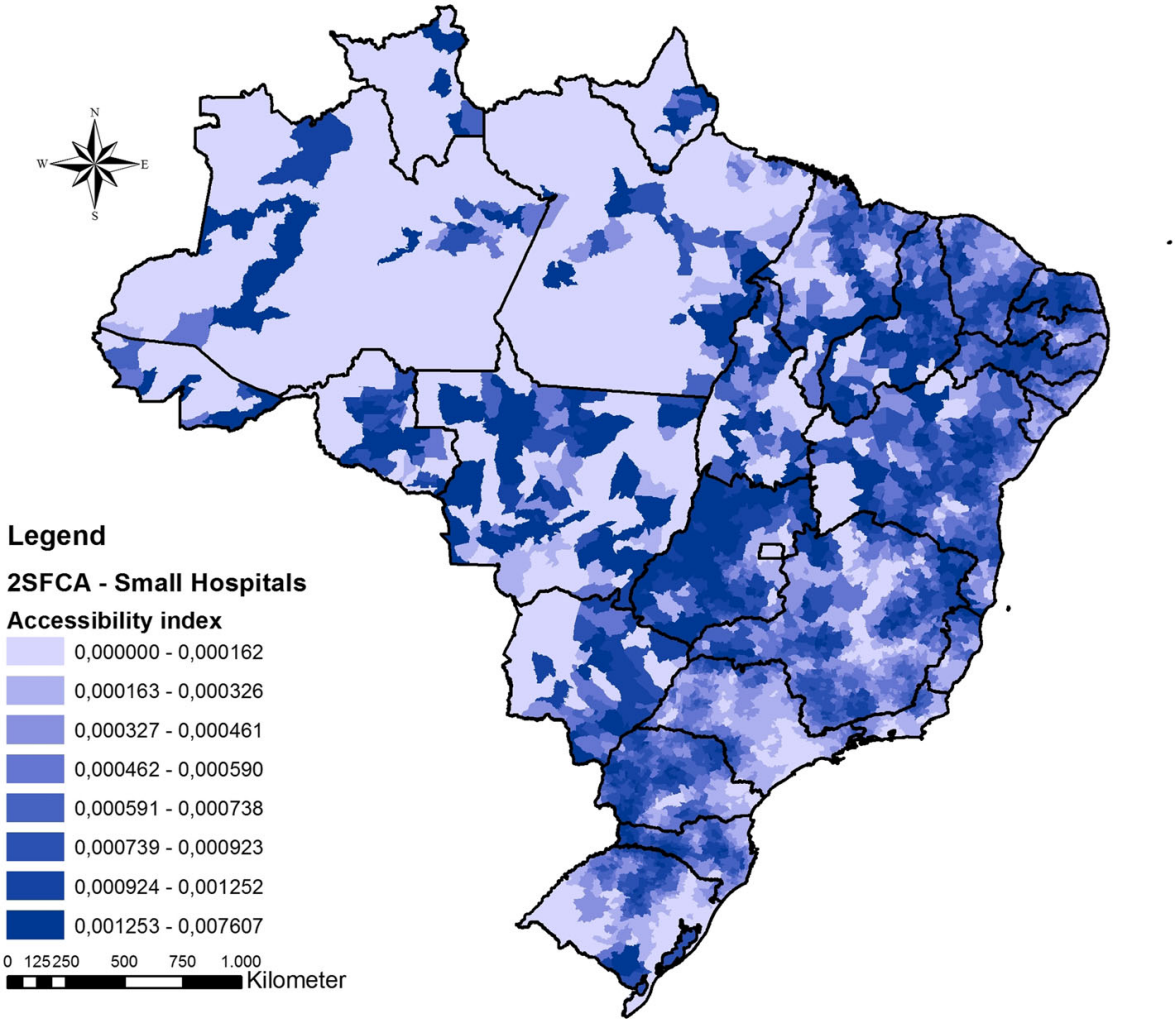
Accessibility index for (a) Small Hospitals in Brazil. Darker colors mean higher cumulative accessibility to hospital beds rate by population

**Fig. 4:**
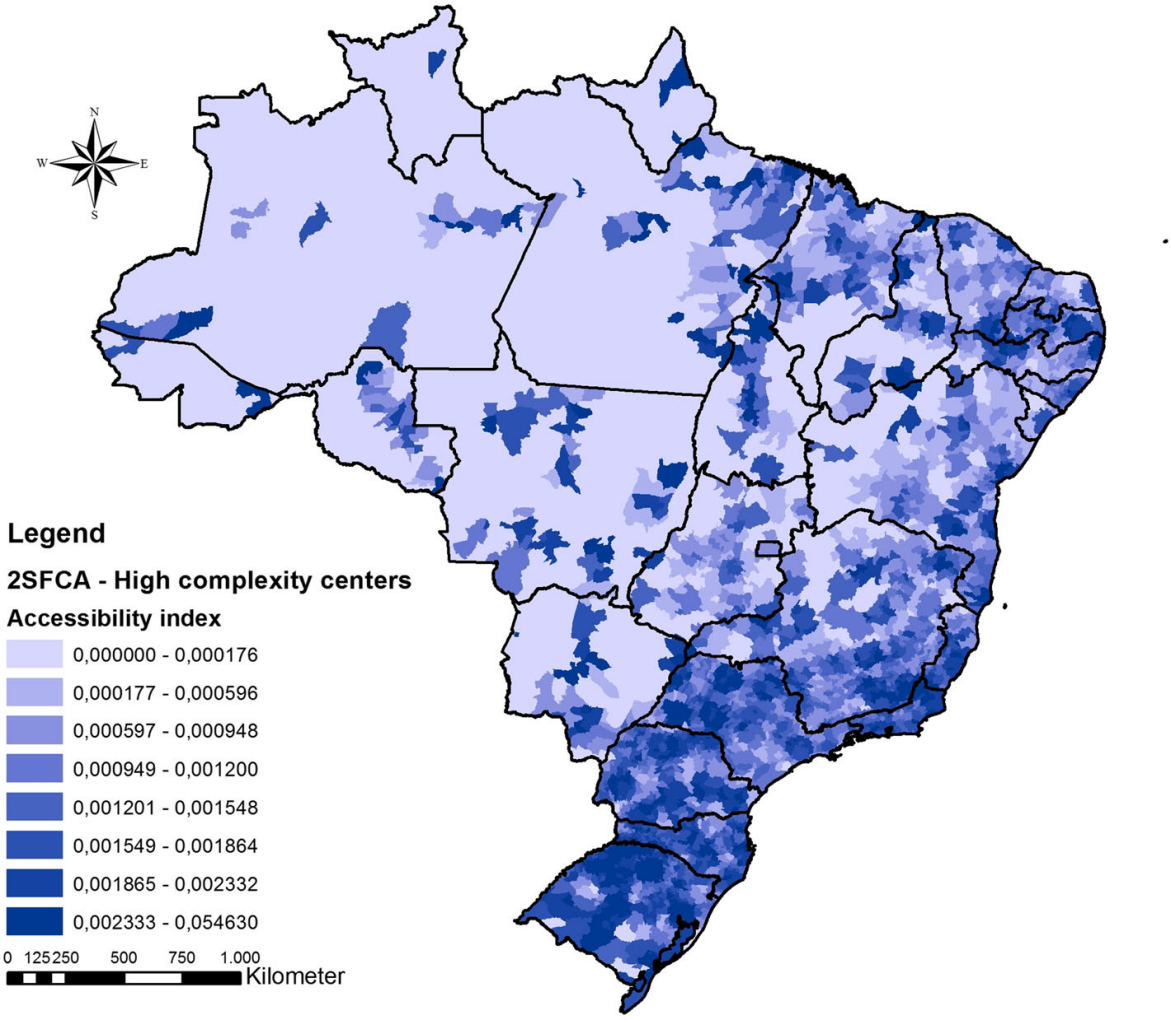
Accessibility index for High Complexity Centers in Brazil. Darker colors mean higher cumulative accessibility to hospital beds rate by population

Although there were states with good accessibility to SH, there was no pattern among regions (Fig. 3). The states of Paraná, Goiás, Minas Gerais, Bahia, Piauí, Rio Grande do Norte, Ceará e Pernambuco presented high levels of accessibility, of hospital beds by inhabitant located within the 60 km catchment area of the SH. For HCC accessibility, there was a concentration of high levels of accessibility in the South, Southeast, and Northeast coastal regions (Fig. 4). The North and Midwest regions experience a shortage of HCC. There were large portions of the country with nearly zero hospital beds by inhabitant considering the HCC accessibility index.

### Small hospitals bridging the gap for emergency surgical care

**Fig. 5:**
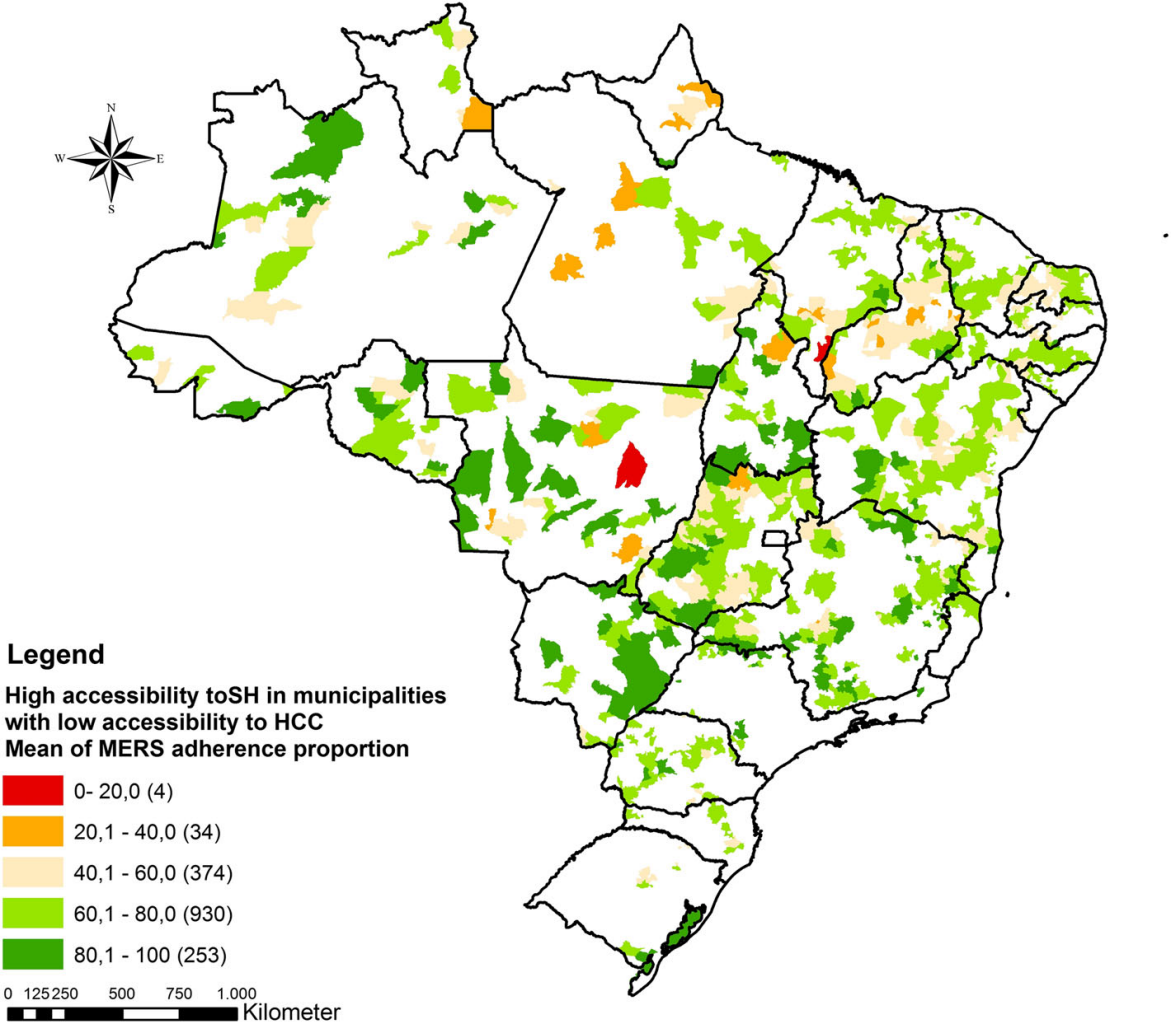
Municipalities with above average access to SH and below average access to HCC considering the minimum emergency service requirements

The ECS care gap analysis of SH and HCC accessibility index showed a group of 1595 municipalities (Table 3) that had high accessibility to SH and low to HCC (Fig. 5). Additionally, to indicate which areas could help overcoming the gap for ECS, the mean scores of SH MESR are depicted in Fig. 5. Considering all municipalities with below average access to HCC, 74% of them (1183) were covered by SH with at least 60% of MESR adherence proportion.

**Table 3:** MERS distribution across Brazilian regions

	Center- West	Northeast	North	Southeast	South	Brazil
Distribution of selected municipalities	268	706	148	342	131	1595
MESR Adherence-mean (SD)	69.13 (15.07)	63.05 (9.86)	67.51 (9.86)	74.76 (10.38)	66.77 (11.86)	67.30 (12.96)

**Fig. 6:**
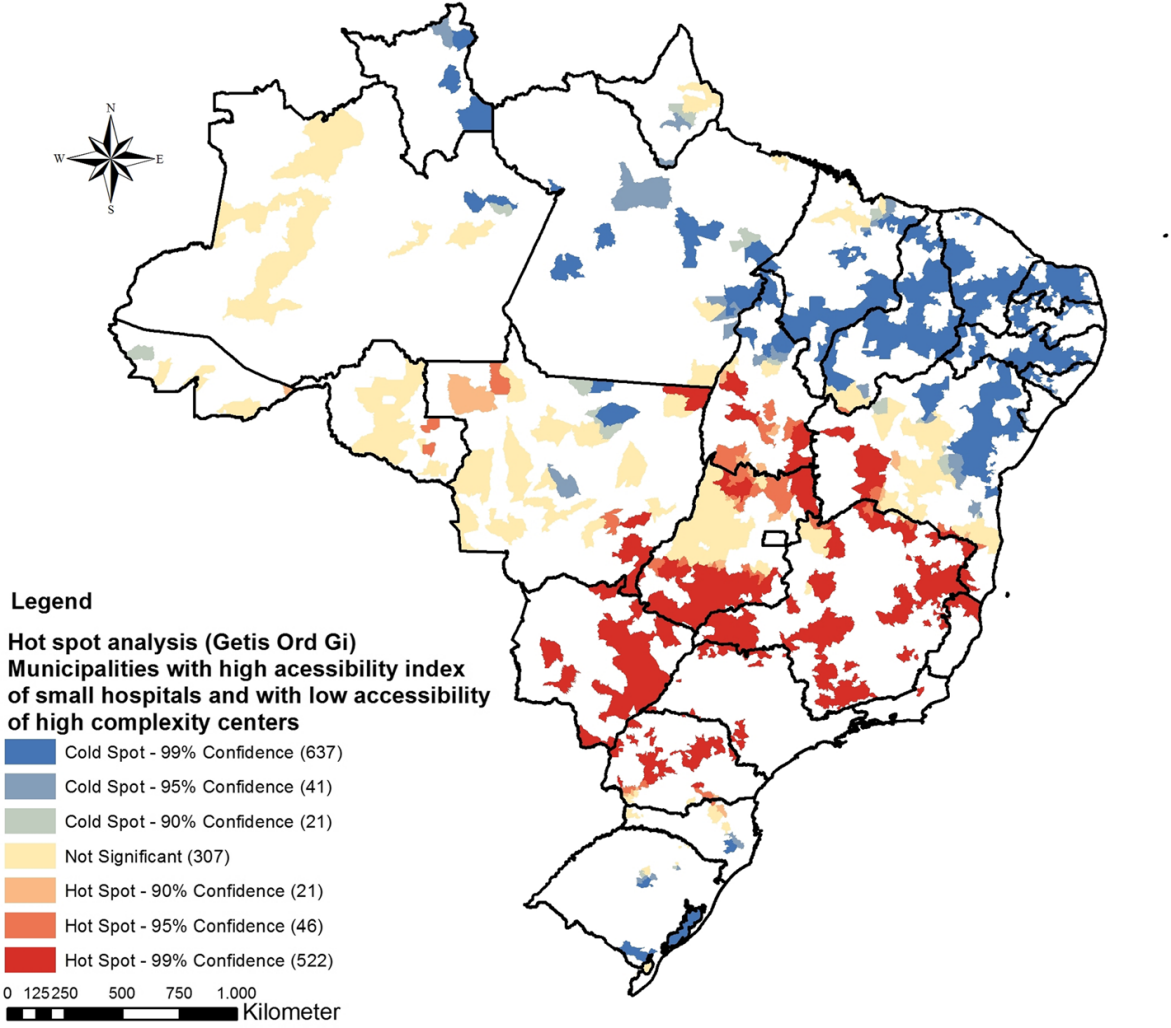
Spatial association of adherence to MESR of municipalities in regions of above average access to SH and below average access to HCC

All municipalities selected after ECS care gap analysis were submitted to a spatial autocorrelation analysis considering its MESR values. Two significant clusters were identified (Fig. 6). The first spatial cluster spans through the Northern and Northeast regions and was comprised of 699 (12,56%) municipalities covered by SH with little ECS capability (blue cold spot). The second significant cluster group was located throughout the Southeast, South and a portion of the Central-West regions. This second cluster was composed of 589 (10,58%) municipalities comprising SH with good ECS capability (red hotspot). This result indicates hotspots areas with SH that meet the criteria to offer surgical care and are, simultaneously, located in regions with lack of access to HCC. The population potentially benefited in these areas comprises more than eight million inhabitants (Table 4). On the other hand, cold spots (blue areas) have SH which are not equipped to match ECS and Surgical care needs, but have enough presence to be a potential solution for the shortage of HCC in those regions. Non-significant areas (yellow) mark locations with dispersed characteristics of SH with and without ECS and Surgical capabilities, filling most of the Center-West region and Amazon area.

**Table 4:** Small hospitals as a possible solution to improve ECS gap of access

	Center- West	Northeast	North	Southeast	South	Brazil
Municipalities classified as ECS Hotspots (% of Brazil)	143 (2.57)	26 (0.47)	33 (0.59)	321(5.77)	66(1.19)	589(10.58)
Population potentially benefited in hotspot areas	2,047,828	575,112	226,881	4,681,838	1,048,436	8,580,095

## Discussion

The present work tried to contribute to diminishing the lack of literature approaching the need for the reduction of geographical access barriers to ECS services. Among the objectives of this effort was the analysis of possible roles to be assumed by Brazilian SH to improve ECS access. In Brazil, one study mapped the network of the provision of health services based on the origin and destination of patients [33]. The results revealed an extensive network of primary care provision, in which only a few municipalities are disconnected. Yet, approximately half of Brazilian municipalities are disconnected from a network of high complexity services. Most SH are found in low and medium sized municipalities. Facilities classified as SH are recognized to face operational and quality problems, despite their prevalence in Brazil [30]. In most cases SH fulfill a role similar to primary care, without the capacity to perform surgeries and admissions [30]. Thus, populations solely reliant on SH frequently need to travel to municipalities with HCC resulting in geographic access barriers.

The analysis of emergency care capabilities among municipalities with SH and those covered by HCC enables the identification of access barriers to high complexity services in Brazil. Furthermore it makes possible an exam of possible roles that SH could assume in the HCN. SH fall short in attaining optimal economies of scale in relation to their function. Therefore, the justification of their existence in HCN service provision is to guarantee access [14, 15]. The results revealed concentrations of municipalities with SH and HCC located in the South, Southeast, and Northeast coastal regions of Brazil. These regions also showed many SH close to reference HCC, which could indicate an overlap among healthcare roles. If some of these small hospitals offer satisfactory emergency care, their role in the HCN is not optimal, and their integration into the health system needs to be rethought. In a large part of north region, there is a gap of ECS services once the emergency units are concentrated in large cities. In these circumstances, the possibility of reconfiguring the role of SH arises as a way of improving access.

SH often experience challenges with lack of structure, human resources, and work processes, and generally do not have a specific role in regional HCN, resulting in idle capacity [2]. One can note the lower coverage of SH in the Central-West, rural Northeast, and, markedly, the North region. States such as Amazonas, Amapá, Pará and Roraima contain large segments of territory without emergency specialized services. The conjoint evidences from 2SFCA and spatial cluster analysis pointed out municipalities covered by SH located in these regions presented low values of MESR adherence proportion. In these circumstances, changing the role of SH is not sufficient. It is essential to invest in the implementation of an integrated HCN that takes into account offering the necessary health services to the population covered. Furthermore analysis about desirable features of each SH should be defined by installed healthcare structure, the composition of the workforce, financial profile and role to be assumed inside a HCN. A combined evaluation of these factors could catalyze reforms to offer better structured ECS.

The situation highlighted by hotspot analysis yields new possibilities to reduce geographical access barriers. Hot spot clusters could be considered as alternatives to increase access to high complexity services. Investments to better outfit SH in these regions could improve access to ECS, as the travel time to a emergency service could be shortened. In these regions SH could get a role dedicated to offer more complex care, contributing to better structuring of HCN. This reorientation can contribute to foster equity in access and strengthen Brazilian emergency services system.

The results demonstrate the distribution of health services in Brazil, based on selected indicators is currently inadequate to meet population needs. Both, the concentration and the role of health facilities in HCN need to be rethought in order to overcome current inequalities in accessing ECS. HCN need to be reorganized in a way that optimizes flows and facilitates efficient processes in economies of scale, without curtailing the population’s access to services [14–16]. The present research revealed that SH could be a possible solution to improve access to ECS, since they receive investments. Fulfilling needs like: better equipment, adequate human resources and defined roles in HCN can create conditions for health facilities provide ECS for a portion of population that is facing access barriers.

While overcoming some limitations of previous studies [34], it is worth emphasizing that distances to health facilities located among states borders were not analyzed which is a limitation of the results found. All analyzed distances were confined to the same state, justifiable by the fact that decentralization in health care has led to the development of policy plans that have an intrastate scope in Brazil. Notwithstanding, there is evidence of agreements among states, primarily in the case of border municipalities, which may influence transportation time to access health services.

For future studies, it would be interesting to exam the services quality offered in emergency facilities as well as adopt dynamically capabilities for each hospital considered in 2SFCA. Added to this contribution should be interesting the evaluation of different indicators, together with parameters from other levels of care, given that reforming of small hospitals roles may be influenced by the quality of care delivered at other levels.

## Conclusions

This study sought to analyze how the spatial distribution of hospitals in Brazil could influence access to ECS, with a focus on the examination of the geographical access barriers. The results demonstrate spatial disequilibrium within the country, with significant gaps in HCN for emergency care and a large concentration of SH in wealthier regions, suggesting imbalances and inequity in service provision. Due to the implications carried out by the spatial distribution of health services and the growing relevance of health geography in health systems design, the current discussion points for the need to reorganize the distribution and roles of hospital network in Brazil. There were several municipalities located greater than 60 km from emergency centers highlighting gaps in emergency coverage that could prove useful to inform policy makers. Although such reorganization may face challenges from an economic and political perspective, the present findings underscore how a combined analysis among different services is necessary to consolidate accessibility and quality in health system.

## Abbreviations

2SFCA: Two-step floating catchment area
ECS: Emergency care services
GDP: Gross domestic product
HCC: High complexity center
HCN: Health care network
LMIC: Low and middle-income countries
MERS: Minimum emergency service requirements
NRHF: National Registry of Health Facilities
SD: Standard deviation
SH: Small hospitals
